# Medical Opinion and Movement

**Published:** 1910-08-06

**Authors:** 


### Medical Opinion and Movement.

### Intratracheal Insufflation of Air.

Some little time back the experimental work of Meltzer and Auer was reported in these columns, by which they had shown that curarised animals could be anæsthetised and kept alive for several hours by a continuous current of air, mixed with ether at a pressure of 15 to 25 millimetres of mercury, conducted through a tube introduced through the larynx into the trachea. It was suggested then that this might prove a practical method of aerisation for carrying out operations on the chest and lungs. Dr. C. A. Elsberg, of Mount Sinai Hospital, New York, has now actually put the method to the test, and reports very satisfactory results. The first case in which the method was tried in the human subject was a woman, aged thirty-four, admitted for myasthenia gravis. Soon after admission respiratory troubles set in, and increased rapidly, so that in twenty-four hours respiratory movements ceased altogether and death appeared imminent. Dr. Elsberg found the patient deeply cyanosed, unconscious, and pulseless. A small tube was introduced into the trachea, and a mixture of air and oxygen insufflated at a pressure of 20 mm. of mercury. The aspect of the patient changed at once. The pulse reappeared, and rapidly rose from 32 to 120. On auscultation the continuous flow of air into the lungs could be heard, and the heart-sounds were clear. The insufflation was continued for five hours, and the physical condition remained good; but the patient did not recover consciousness. Encouraged by this observation, Dr. Elsberg then applied the method to a case of abscess of the middle lobe of the right lung. The pharynx and larynx were swabbed with 10 per cent. cocaine solution and a tube was then introduced nearly as far as the bifurcation of the trachea. A mixture of ether and air was then introduced at a pressure of 15 mm. of mercury. The patient was placed on the left side and the operation carried out, lasting three-quarters of an hour, without any trouble. The patient made an excellent recovery. Since then Dr. Elsberg has again employed the method successfully in a third case of empyema following pneumonia. He expresses the belief that this method of continuous intratracheal insufflation of air will prove of valuable assistance in intrathoracic operations, and will minimise the dangers attending this branch of surgery.

### New Treatment of Vesical Neoplasms.

New growths of the bladder rank among the most rebellious to surgical treatment. The benign, as well as the malignant, are likely to prove eventually fatal if untreated, and, on the other hand, removal by the surgeon's knife more often than not is followed by recurrence. Removal by the suprapubic method is so frequently followed by rapid recurrence in the suprapubic wound that it has been generally condemned and abandoned in favour of the operating cystoscope. This method, first conceived by Civiale, has been elaborated and developed by Nitze; but it requires great skill and technical knowledge, and cannot be said satisfactorily to solve the problem. Great interest, therefore, will be aroused by the new method devised by Dr. E. Beer, of the Mount Sinai Hospital, New York, by which he seeks to remove vesical tumours by the application of the high-frequency current to the growth through an ordinary cystoscope. A Nitze double catheter cystoscope is used. He employs the Oudin or single pole high-frequency current, with a spark gap of one-tenth to an eighth of an inch. In one catheter tunnel he places the electrode, while to the other he attaches a tube for irrigation. The electrode is a simple six-ply cable of copper wire, insulated with rubber and cut off squarely at the vesical end. It corresponds to No. 6 French. The applications are made directly to the growth, the electrode being pushed a short distance in among the villi under the guidance of the eye, and the current turned on for 15 to 30 seconds at various points. The bladder is distended with distilled water. Gas is freely generated at the growth, and the tissues are blanched, and at the point where the electrode was placed the tissues are blackened. The tumour may come away with the electrode firmly baked to its point. Bleeding is easily checked by a reapplication of the current. No more discomfort is caused than in an ordinary cystoscopy. Beer ascribes the necrosis of the growth to the heat, though other factors—ionisation, electrolysis—probably contribute. He has carried out the treatment successfully in two cases of large papillary growths, and thinks the treatment has a wide field of usefulness. Immediately following this publication in the *Journal of the American Medical Association*, Professor E. L. Keyes, of New York, also reports on the treatment in the *American Journal of Surgery*. He has employed it in five cases. Three of these were malignant and in advanced stages, and no definite good resulted; but in two cases of papilloma he has also obtained satisfactory results by the treatment.

### Anaphylaxis.

Since the clinical and experimental phenomena of anaphylaxis were clearly established, a good deal of research work has been carried out to discover a means of preventing their occurrence after serum injections in the human subject. Drs. Carnot and Slavu think they have discovered a method by adding a certain quantity of hydrochloric acid to the serum before injection. They find experimentally that the addition of 3.3 per 1,000 hydrochloric acid to the serum is sufficient to prevent the occurrence of anaphylaxis in guinea-pigs that have been previously rendered susceptible, and this amount of acid is not in any way injurious to the antitoxic properties of the serum. Dr. Besredka adopts a more rational and physiological method by which he seeks to render the subject immune. He finds experimentally that a guinea-pig previously anaphylactised passes rapidly into a condition of anti-anaphylaxis after an injection of a small dose (0.05 c.c.) of serum. This small dose acts as a vaccine, and afterwards fatal, or even twice fatal, doses can be injected without any effect. This immunity is acquired about three hours after subcutaneous and almost instantaneously after intravenous injection. The author points out the value of this rapidity with which the vaccinating injection establishes immunity, as by a series of injections in increasing doses complete immunity can be obtained in a very short time. In the human subject in cases in which large doses of serum are to be injected, the author proposes that they should be given successively at short intervals and in increasing doses. In this way he has succeeded in obtaining complete immunity in guinea-pigs which resisted every test.

### Electrical Injuries.

In the *Journal of the American Medical Association* Dr. Morehead, of New York, after drawing attention to the scantiness of the literature on the subject, discusses injuries caused by electricity. He maintains that with a voltage of from 200 to 500, dangerous injuries are not usually caused, though the alternating is considered more dangerous than the direct current. High voltage and contact for long periods of time are, of course, very injurious. If the voltage be of medium intensity, and contact only partial and for a short period of time, burns of the first and second degree, with moderate degrees of coma and shock and a certain amount of paraesthesia are the result. Paraesthesia of the formication type is caused by still slighter intensity and a brushing contact. The higher the amperage is, the greater the result, and there does not seem to be much difference in the effects caused by the site of contact, or its area. The resistance of the tissues would seem to vary in proportion to their fluid content, the order of conductivity of the tissues being, blood, muscle, nerve, and bone. Males are usually more resistant than females, and individuals differ from one another in respect of conductivity. Death from electricity usually occurs suddenly and leaves some sign, such as burns, whereby the cause can be recognised, but gross changes in the body are not as a rule met with. The burns only differ from those caused by heat in being somewhat less painful, in producing less shock, and in healing more quickly. Treatment is mainly directed towards the relief of symptoms, but prolonged artificial respiration should always be used in cases of apparent death from electricity. Prognosis depends on the length of the duration of the initial symptoms of inhibition, and to some degree on the amount of septic and toxic products absorbed from the burns.

### The Mechanism of the Menstrual Cycle.

In the *Medical Record* Dr. Loeb publishes a further instalment of his investigations into the functions of the corpus luteum, the experimental production of a placenta, and the mechanism of the sexual cycle in the female. This embodies his researches since his last paper of a year ago, and is based upon the examination of more than nine hundred guinea-pigs and of a large number of rabbits. He concludes that the corpus luteum has an influence which acts as a formative stimulus upon the mucosa of the uterus and induces the phenomena of growth. When in addition to this luteal influence a mechanical stimulus to the mucosa is present, growth proceeds much further and leads to placenta formation. This stimulus is provided in the ordinary phenomenon of pregnancy by the ovum, but other experimental objects can be substituted and thus a placenta artificially produced. The corpus luteum is an essential part of the process, and reasons are advanced for the belief that an internal secretion is produced in it which the rest of the ovary is incapable of supplying. Also the continued supply of this internal secretion is necessary, for if it be cut off by removal of the corpus luteum the developing placenta fails to attain full growth. After the introduction of foreign bodies into the uterus the newly formed decidual tissue often far exceeds the quantity of placenta produced in a normal pregnancy. Furthermore, the corpus luteum prolongs the sexual cycle, the interval between successive ovulations, by preventing rupture of the ovarian follicles; the growth of the embryo in the absence of the corpus luteum has no such effect. The author has not yet discovered what factor it is which extends the life of the corpus luteum when conception has taken place.

### Brill's Disease.

In a critical survey of the diseases which may simulate typhoid fever Dr. Ziegel, of New York, pleads for the recognition of the separate clinical entity which Brill has been describing for the last fourteen years as a disease clearly separable from typhoid. It is no exaggeration, he maintains, that the clinical differences between these two conditions are so pronounced and easy of recognition that their differentiation is easier than that of German measles from measles and scarlet fever. In Brill's disease the eruption appears as a single crop usually on the seventh day of the illness. It appears first on the abdomen and back, spreading rapidly to the chest and extremities; it may appear on the palms and soles, and is often more profuse on the arms than on the trunk. The spots are maculo-papular, and usually have an oval indistinct outline; they do not quite entirely disappear on pressure. Occasional hæmorrhagic spots may be seen, and also others very like the true typhoid roseola. The invasion of the disease is fairly abrupt, and may be accompanied by chilly sensations, severe headache, and high fever; general malaise usually precedes these symptoms. The temperature reaches its acme in about three days and then remains sustained until the rather rapid lysis. While the fever lasts the headache persists severe, and in a small proportion of cases there is also meningismus. Apathy and prostration are marked, and the patient looks as ill in the first week as does a typhoid patient in the second or third. After one or two weeks of illness the temperature falls to normal in from ten to sixty hours, and the symptoms rapidly ameliorate. Relapses and serious sequelæ are rare; the mortality is nil. Nothing is known of the pathogenesis of this disease; it has been thought to be possibly mild typhus, but Brill is strongly opposed to this idea. The disease does not occur in epidemics, and it is but little communicable from one patient to another.

### Radium Emanations.

In the *Berliner Klinische Wochenschrift* there appears a paper from the pen of W. Engelmann on the use of radium emanations by means of baths, packs, and local applications. He carried out a series of observations for the purpose of demonstrating that the efficacy of such treatment was due to the effects of absorption through the skin directly. Baths of the emanations were given to healthy individuals after taking care that their heads were protected from the action of the rays. The air expired by these test cases was carefully examined for radio-activity, both during and after bathing, and in every case emanations were discovered in the breath, their amount being directly proportional to the length of the exposure of the individuals to the radio-active baths.

### Ruptured Uterus.

An extraordinary case of intra-partum spontaneous rupture of the uterus, due to absence of the vagina, has been reported to the New York Academy of Medicine by Dr. Louis J. Ladinski. The patient, aged twenty-three, had been married eleven months. Her past history was that menstruation had started at fifteen, after a specialist had performed an operation upon her of which she did not know the nature. In March of this year she was admitted to hospital and examined. Physical examination revealed all the signs of a full-term pregnancy with the fœtus in the normal position, the vertex presenting. About half an inch above the introitus vaginæ there was a thick septum in the centre of which was an opening about one-quarter of an inch in diameter. The fœtal head could be distinctly felt pressing against this septum. The patient was prepared for a thorough examination under an anæsthetic on the day following, but during the night labour pains commenced, and before the author could reach the hospital she collapsed, became cyanotic, and her pulse rapid and uncountable. The fundus uteri could not be felt, the epigastrium was bulging, tense, and dull to percussion. The fœtal mass could be felt just beneath the abdominal wall. At the laparotomy which was performed immediately the peritoneal cavity was found to be distended with a large quantity of blood and clots. The body and extremities of the fœtus extended through a rent in the anterior uterine wall, the head being grasped by the contracting ring of the lower uterine segment and cervix. On withdrawing the fœtus the placenta followed. The uterus was low down in the pelvis, hard and contracted, with a complete transverse rent at the level of the junction of the body and cervix, extending through the entire anterior wall, and embracing part of the posterior wall, so that only a narrow isthmus attached the cervix to the uterus. The uterus was removed entire. No vagina could be found, the cervix uteri extending down to the introitus vaginæ.

### Helminthiasis in Children.

According to the *American Journal of the Medical Sciences*, a routine examination of the fæces of a large number of children in New York City has been made by Dr. Schloss. Worms were found in 12 out of 30 children suffering from obscure nervous and gastro-intestinal symptoms. The parasites were also discovered in 80 out of 280 consecutive cases examined. The following is a list of the varieties of worms found, together with the number of cases of each: Trichiuris trichiura 31, Oxyuris vermicularis 23, Hymenolepsis nana 20, Ascaris lumbricoides 6, and Tænia saginata 5. In 35 of the 51 children suffering from other parasites symptoms were complained of. Examination of the blood showed that cases complaining of symptoms of helminthiasis were possessed of eosinophilia except when these were due to the Trichiuris trichiura, when there was no increase of eosinophile cells. When, however, no symptoms were complained of, the presence of parasites had no effect on the nature of the blood cells present.

### Mosquito Traps.

According to *L'Hygiène générale et appliquée* a French officer in Africa observed mosquitoes issuing at sunset from crab-holes in the ground, and from that fact conceived the idea of constructing artificial holes of a similar nature to trap the insects. The mouth of the hole, an inch in diameter and rectangular in shape, leads into channels some 18 inches deep, forming an acute angle with the surface. The mouth of the hole is so disposed as to be sheltered from the wind and sun. These traps were found to be highly successful, and it is suggested that wooden imitations might prove useful for indoor use.

### Sudden Death from Suppurating Thymus.

Planchu and Rendu, according to *La Clinique*, have lately reported the case of an infant of five months which suddenly, while apparently in good health, was attacked by extreme cyanosis from which it only recovered after being rubbed, poulticed, and artificially respired for a quarter of an hour. A second attack a month later, which occurred without any prodromal symptoms, carried off the child. At the autopsy typical acute œdema of both lungs, together with a hypertrophied and suppurating thymus, were found. Histological examination of this last organ showed it to be infiltrated with little miliary abscesses. The authors believe that the acute œdema of the lungs was brought on by one of two causes, either by the hypertension resulting from the abolition of the internal secretion of the gland, or by the excitation of the terminal branches of the vagus caused by the inflamed organ. They point out that, whatever be the true reason, the thymus should always be carefully examined, even if there appear to be pulmonary lesions present sufficient to cause death. Many lesions of this gland must have escaped notice in the absence of a proper histological examination.

